# The Clot Thickens: A Shocking Case of Multivessel Arterial Embolism Despite Therapeutic Anticoagulation

**DOI:** 10.51894/001c.144437

**Published:** 2025-09-30

**Authors:** Michael Collins

**Affiliations:** 1 Department of Internal Medicine Henry Ford Wyandotte

## 72

### INTRODUCTION

Atrial fibrillation increases the risk of thromboembolism, commonly mitigated with anticoagulation such as warfarin. Despite a therapeutic INR, thrombus formation remains a rare but devastating possibility. We present a case of a patient on warfarin with a therapeutic international normalized ratio (INR) who developed multiple arterial emboli, ultimately resulting in death.

### CASE REPORT

This unique case report describes a 64-year-old man with atrial fibrillation, CHF, type 2 diabetes, hypertension, and PAD s/p left BKA who presented to the emergency department with complaints of generalized body aches and dizziness. While on telemetry, the patient was noticed to have runs on ventricular tachycardia. Once stabilized, the patient additionally complained of lower back pain radiating to the abdominal pain, malaise, chills, and shortness of breath. Given concerns for aortic dissection, computed tomography angiography (CTA) of the chest, abdomen, and pelvis was performed, and interpreted by the radiologist as significant for right sided obstructive uropathy, cholelithiasis, and a left atrial thrombus, but no evidence of dissection.

Shortly after returning from imaging, the patient began complaining of right leg numbness and weakness. On re-evaluation, his right leg was cold and pulseless below the proximal femoral artery. Vascular surgery initially planned for thrombectomy but identified an acute SMA occlusion not seen by the radiologist, necessitating transfer for higher-level intervention. After extensive discussions regarding prognosis, including potential amputation of his remaining leg and colostomy, the patient opted for hospice care and passed away 48 hours later.

### DISCUSSION/CONCLUSIONS

This case underscores the need for emergency physicians to maintain a high index of suspicion for arterial thromboembolism, even in anticoagulated patients with a therapeutic INR. Despite appropriate warfarin therapy, this patient developed simultaneous life-threatening thromboembolic events- acute lower extremity arterial occlusion and acute mesenteric ischemia. Additionally, initial imaging interpretation missed critical findings, emphasizing the importance of independent image review by emergency physicians. Timely recognition and intervention are crucial, as delayed diagnosis can lead to irreversible complications and poor outcomes.

